# HIV Prevalence by Race Co-Varies Closely with Concurrency and Number of Sex Partners in South Africa

**DOI:** 10.1371/journal.pone.0064080

**Published:** 2013-05-21

**Authors:** Chris Kenyon, Jozefien Buyze, Robert Colebunders

**Affiliations:** 1 HIV/STD Unit, Institute of Tropical Medicine, Antwerp, Belgium; 2 Division of Infectious Diseases and HIV Medicine, University of Cape Town, Cape Town, South Africa; 3 Department of Clinical Sciences, Institute of Tropical Medicine, Antwerp, Belgium; 4 Clinical HIV/STI Unit, Institute of Tropical Medicine, Antwerp, Belgium; Vanderbilt University, United States of America

## Abstract

**Background:**

HIV prevalence differs by more than an order of magnitude between South Africa's racial groups. Comparing the sexual behaviors and other risk factors for HIV transmission between the different races may shed light on the determinants of South Africa's generalized HIV epidemic.

**Methods:**

Five nationally representative and one city-representative population-based surveys of sexual behavior were used to assess the extent to which various risk factors co-varied with HIV prevalence by race in South Africa.

**Results:**

In 2004, the prevalence of HIV was 0.5%, 1%, 3.2% and 19.9% in 15–49 year old whites, Indians, coloureds and blacks respectively. The risk factors which co-varied with HIV prevalence by race in the six surveys were age of sexual debut (in five out of five surveys for men and three out of six surveys for women), age gap (zero surveys in men and three in women), mean number of sex partners in the previous year (five surveys in men and three in women) and concurrent partnerships (five surveys in men and one in women). Condom usage and circumcision were both more prevalent in the high HIV prevalence groups. The reported prevalence of concurrency was 6 to 17 times higher in the black as opposed to the white men in the five surveys.

**Conclusions:**

The differences in sexual behavior in general, and the prevalence of concurrency and the number of sexual partners in particular, offer a plausible and parsimonious cause to explain a part of the differing prevalences of HIV between South Africa's racial groups.

## Introduction

In 2004 adult HIV prevalence varied by a factor of 40 between South Africa's racial groups – 0.5%, 1%, 3.2% and 19.9% in 15–49 year old whites, Indians, coloureds and blacks respectively [1]. Since these populations are exposed to the same circulating HIV subtypes, the most plausible explanatory factors for this variation are differences in sexual behavior, barrier contraception usage, circumcision prevalence, host genetic susceptibility and the prevalence of other STIs. Only one published study has investigated the extent to which these risk factors could explain HIV's differential spread by race in South Africa [2]. This study found that network-level sexual behaviors were the most plausible cause but it was limited by being based on a dataset that was only representative of 14–22 year olds from the city of Cape Town. In this study we use the data from five nationally representative adult and youth surveys in addition to the same youth survey from Cape Town to evaluate the extent to which each of these risk factors co-varies with HIV prevalence by racial group.

## Materials and Methods

The methodologies of the six surveys used have been described in detail elsewhere but are summarized below [1], [3], [4], [5], [6], [7]. The 1998 South Africa Demographic Health Survey (DHS) was a 2-stage sample in which South Africa's 9 provinces were stratified into urban and nonurban groups. It was designed to be representative for the 9 provinces, urban versus rural areas and the four major racial groups. The survey was conducted on 11735 15- to 49-year-old women. Although men were included as a further sample, they were not asked questions pertaining to sexual behavior. The overall response rate was 92.3%. The point prevalence of concurrency was derived from the question: “Do you currently have a regular sexual partner, an occasional sexual partner, or no sexual partner at all?” Individuals' responses were coded into four categories: “regular sexual partner, two or more regular partners, occasional sexual partner and no sexual partner.” The individuals coded as having two or more regular partners were classified as concurrents and all others as non-concurrents.

The 2003 DHS used a similar two-stage sampling methodology to sample 7966 15–49 year old women. In addition, a smaller number of households were sampled to recruit 3930 men of the same age. The overall response rates for the women and men were 74.7% and 67.8% respectively.

In the first South African National HIV Prevalence, HIV Incidence, Behavior and Communication Survey (2002 SABSSM), 9963 males and females aged 2 and above were sampled. The survey used a multi-stage stratified sampling approach. When correctly weighted to account for the sampling design and HIV testing non-response, the sample was representative of the population in South Africa for the main reporting domains of sex, age, race and province. The overall response rate was 74.0%. Our data analysis was limited to the 7774 persons aged 15 to 49.

The second SABSSM (2005) survey used a broadly similar sampling method to its predecessor. The survey had an overall response rate of 80.7% and the sample consisted of 23275 persons 2 years old or older. We limited our analysis to the 13,884 individuals aged 15 to 49 years old.

The National Communication Survey (NCS) was a cross-sectional survey that utilized a three-stage, stratified sampling approach. The survey produced a nationally representative sample of 9728 individuals aged 16 to 55 in 2009. The overall response rate was 58%. We limited our analysis to the 9026 individuals aged 16–49 years old. For further details of the methodology and possible bias introduced by differential non-response see Johnson et al. [4] For the DHS 2003, SABSSM 2002, SABSSM 2005 and the NCS, the point prevalence of concurrency was defined dichotomously based on the question – how many sexual partners do you currently have. All individuals reporting two or more current partners were coded as concurrents.

The Cape Area Panel Study (CAPS) was a representative longitudinal study of adolescents, aged 14 to 22 (in 2002) living in Cape Town, conducted in five waves. It used a two-stage probability sample of households. Waves 3 and 5 included modules on sexual activity. The response rate for whites was very low in wave 5 and thus we elected to use wave 3. This wave was conducted in 2005 and its overall response rate was 75%. Respondent concurrency was measured via the question ‘Did you have any other sexual partners during the time that you and [partner number 1–10] were having a sexual relationship?’ If the respondent indicated “definitely yes” to this question for any of their previous ten sexual relationships they were coded as concurrent. All the other individuals were coded as non-concurrent. Concurrency was thus defined as the point-prevalence of concurrency at the time of the survey for all the surveys except for the CAPS where concurrency was defined as the cumulative prevalence of concurrency. UNAIDS recommends the use of both point- and cumulative-prevalence of concurrency in the evaluation of concurrency [8]. It should be noted that the cumulative prevalence of concurrency as measured by the CAPS would be expected to be higher than the point-prevalence of concurrency as measured by the other surveys.

The participants in each of these surveys were asked to self-identify with one of four racial categories: black (African), coloured, white and Indian. The term “coloured” is a common and socially acceptable term in South Africa for individuals of mixed race. This research involved secondary data analysis of six surveys who had each received ethical committee clearance for data analyses such as the one performed here [1], [3], [4], [5], [6], [7]. All data is aggregated to the level of communities and thus anonymity is preserved. No specific ethics committee approval was necessary for this study.

### Statistical analyses

All analyses were made using STATA 12.0 (College Station, TX). Allowance was made for the complex sampling strategies of the six surveys using the survey (SVY) methodology.

This provided population-based prevalence estimates of behaviors. The analyses were stratified by gender. Pearson χ^2^ tests were used to compare categorical variables and Student's T-tests were used to compare the means of continuous variables. All comparisons were limited to within survey comparisons. In all cases the comparisons were between each racial group and the black group – the group with the highest HIV prevalence. All tests were performed at a significance level of 1.6% or 2.5% as a Bonferroni correction for multiple comparisons - three in all the surveys except CAPS where there were only two comparisons. All individuals 15–49 years old were included in the analyses for age and sexual experience. For all the other variables the analyses were restricted to those who had had sex.

## Results

### Demographics

**Figure 1:**
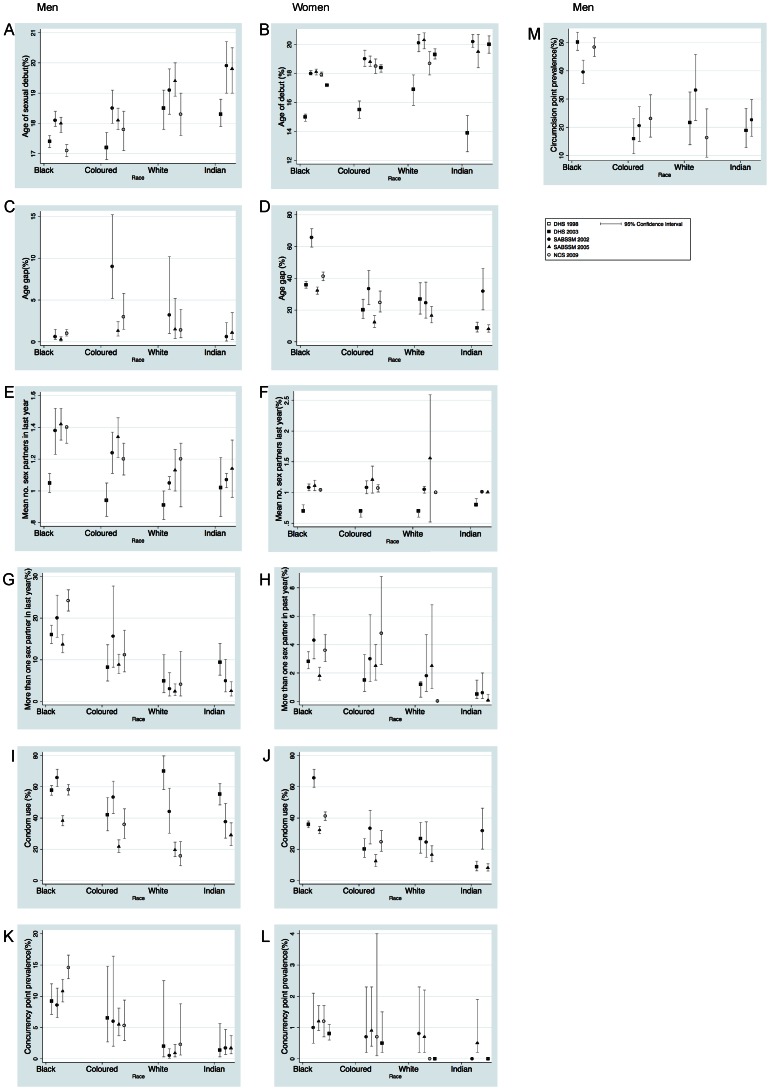
Prevalence of sexual behaviors, condom use and circumcision by race and sex in five South African surveys of 15–49 year olds. Mean age of sexual debut (A,B), Age gap – the percentage of respondents with a partner five (ten in the case of DHS 2003) or more years older than them (C,D), Mean number of sex partners in the past year (E,F), Percent of respondents who had more than one sex partner in the previous year (G,H), Percentage respondents who used a condom at last sex (I,J), Percent respondents who reported concurrent relationships at the time of the interview (K,L), Percent of men who reported having been circumcised (M). Men and women represented in the left and right hand columns respectively (Point estimates with 95% Confidence Intervals. A,B and M refer to all respondents and C–L to those who have had sex). DHS (Demographic and Health Survey) 1998 and 2003, SABSSM (South African National HIV Prevalence, HIV Incidence, Behavior and Communication Survey) 2002 and 2005, NCS (National Communication Survey) 2009.

For both men and women, the black group was significantly younger than the other groups in each of the surveys except the CAPS where the opposite was the case (see Tables 1,2 and Figure 1). Where age differences occurred, they were generally less than two years.

**Table 1:** Prevalence of sexual behaviors, condom use and circumcision by race in three South African surveys – CAPS (Cape Area Panel Survey) 2005 and DHS (Demographic and Health Survey) 1998 and 2003.

	CAPS 2005			DHS 1998				DHS 2003			
Men	Black	Coloured	White	Black	Coloured	White	Indian	Black	Coloured	White	Indian
**n**	836	897	181					2327	348	156	282
**Age – mean years (95% CI)**	21.2 (21.0–21.4)	20.7 (20.5–20.9)***	20.3 (19.9–20.6)***					31.08 (30.53–31.63)	33.43 (31.67–35.24)	35.43 (32.57–38.30)**	36.18 (34.29–38.08)***
**Ever had sex - % (95% CI)**	86.2 (83.5–88.4)	66.8 (62.5–70.9)***	49.5 (39.8–59.1)***					81.5 (79.5–83.4)	79.7 (71.7–85.9)	84.3 (76.8–90.0)	82.9 (75.0–88.7)
**Age of debut - mean years (95% CI)**	15.5 (15.3–15.8)	16.4 (16.1–16.6)***	17.5 (16.9–18.1)***					17.4 (17.2–17.6)	17.2 (16.8–17.7)	18.5 (17.8–19.1)**	18.3 (17.9–18.8)***
**% with more than one sex partners in last 12 months (95% CI)** a	33.4 (29.7–37.3)	19.7 (15.9–24.1)**	14.2 (7.0–26.4)**					16.0 (13.9–18.3)	8.2 (4.9–13.6)*	4.9 (2.1–11.2)**	9.4 (6.3–13.9)*
**Mean/median no. sex partners in last 12 months (95% CI)** a	1.4/1 (1.3–1.5)	1.2/1 (1.1–1.3)**	1.1/1 (0.9–1.3)**					1.05/1 (0.99–1.11)	0.94/1 (0.84–1.05)	0.91/1 (0.82–1.00)	1.02/1 (0.84–1.21)
**Mean no. lifetime sex partners (95% CI)** a	2.9 (2.7–3.1)	2.7 (2.3–3.1)	2.6 (1.5–3.6)								
**Age gap -% (95% CI)** a **^,^** b	7.8 (5.8–10.5)	6.0 (4.2–8.5)	5.1 (1.5–16.3)								
**Concurrency - % (95% CI)** a	34.2 (30.6–38.1)	13.4 (10.8–16.5)***	2.1 (0.5–7.8)***					9.2 (7.1–12.0)	6.5 (2.7–14.8)	2.0 (0.3–12.5)	1.4 (0.3–5.6)**
**Condom use - % (95% CI)** a	79.2 (74.8–82.9)	61.6 (56.4–66.5)***	80.4 (63.3–90.7)					57.7 (54.6–60.8)	42.1 (31.8–53.1)**	70.1 (58.2–79.8)	55.3 (48.4–62.1)
**Circumcision - % (95% CI)** e	92.2^e^	32.0	18.0					50.2 (47.3–53.7)	15.9 (10.6–23.0)***	21.7 (13.8–32.5)***	18.8 (12.8–26.7)***
**Women**											
**n**	683	783	156	8993	1533	755	393	5234	933	274	596
**Age – mean years (95% CI)**	21.2 (21.0–21.4)	20.7 (20.5–20.9)***	20.5 (20.1–21.0)**	29.1 (28.9–29.4)	30.1 (29.5–30.8)**	32.1 (31.2–33.1)***	31.2 (30.3–32.0)***	29.35 (29.05–29.64)	30.49 (29.84–31.14)**	32.87 (31.59–34.15)***	32.55 (31.49–33.61)***
**Ever had sex - % (95% CI)**	89.7 (86.7–92.0)	59.9 (55.7–64.0)***	57.4 (49.1–65.3)***	88.5 (87.5–89.3)	81.2 (78.5–83.7)***	80.6 (76.4–84.3)***	75.2 (71.1–78.9)***	82.0 (80.6–83.4)	79.4 (75.6–82.7)	85.0 (78.4–89.9)	53.0 (46.1–59.7)***
**Age of debut - mean years (95% CI)**	16.5 (16.4–16.7)	17.5 (17.3–17.7)***	17.4 (16.9–17.9)***	17.2 (17.1–17.3)	18.4 (18.1–18.6)***	19.3 (19.0–19.7)***	20.0 (19.4–20.6)***	15.0 (14.7–15.2)	15.5 (14.9–16.1)	16.9 (15.8–17.9)***	13.9 (12.6–15.1)
**% with more than one sex partners in last 12 months (95% CI)** a	9.3 (7.5–11.6)	2.8 (1.5–4.9)***	10.3 (4.9–20.3)	4.2 (3.7–4.8)	1.7 (1.0–2.9)**	1.6 (0.7–3.4)**	0.4 (0.05–2.4)**	2.8 (2.3–3.5)	1.5 (0.7–3.3)	1.2 (0.3–1.4)	0.5 (0.2–1.5)***
**Mean/median no. sex partners in last 12 months (95% CI)** a	1.05/1 (1.02–1.08)	0.94/1 (0.9–0.97)***	1.16/1(1.03–1.29)	1.03/1(1.02–1.04)	1.01/1 (0.97–1.05)	1.02/1(0.99–1.05)	0.99/1 (0.95–1.03)	0.7/1 (0.7–0.8)	0.7/1 (0.6–0.7)	0.7/1 (0.6–0.7)	0.8/1 (0.8–0.9)
**Mean no. lifetime sex partners (95% CI)** a	2.3 (2.2–2.4)	1.5 (1.4–1.6)	2.4 (1.9–2.8)								
**Age gap -% (95% CI)** a **^,^** b	31.0 (27.7–34.4)	27.6 (23.4–32.2)	13.1 (6.9–23.3)**					9.8 (7.7–12.3)	7.5 (3.5–15.3)	13.5 (4.7–32.8)	6.4 (0.9–33.3)
**Concurrency -% (95% CI)** a	15.9^d^ (13.4–18.7)	1.7^d^ (0.9–2.8)***	2.7^d^ (1.0–7.2)***	0.8^c^ (0.6–1.1)	0.5^c^ (0.2–1.5)	0^c^	0^c^				
**Condom use -% (95% CI)** a	72.4 (68.8–75.8)	39.0 (33.6–44.6)***	89.1 (75.0–95.8)	12.6 (11.6–13.7)	7.9 (6.0–10.5)**	9.6 (6.8–13.4)	6.5 (4.0–10.4)**	35.9 (33.8–38.1)	20.1 (14.8–26.8)***	26.2 (17.5–37.2)	8.8 (6.2–12.4)***

* *P*<0.01, **P<0.001, ***P<0.0001 (The *P*-values refer to comparisons between the each racial group and the black group within that same survey.

a These analyses are limited to the individuals who are sexually experienced.

b Age-gap refers to the respondents most recent sexual partner being 5 or more years older than the respondent. The only exception was the DHS 2003 survey where this gap was measured as 10 years.

c The proportion reporting concurrent relationships in the DHS 1998 is limited to those who are unmarried.

d Concurrency in the CAPS is a cumulative prevalence of concurrency in any of the last 10 sexual relationships as detailed in the methods section.

e Circumcision prevalence was first measured in Wave 5 of CAPS. The prevalences reported here therefore refer to the males aged 20–30 years old who reported that they had been circumcised [45], [46].

**Table 2:** Prevalence of sexual behaviors, condom use and circumcision by race in three South African surveys – SABSSM (South African National HIV Prevalence, HIV Incidence, Behavior and Communication Survey) 2002 and 2005, NCS (National Communication Survey) 2009.

	SABSSM 2002				SABSSM 2005				NCS 2009			
Men	Black	Coloured	White	Indian	Black	Coloured	White	Indian	Black	Coloured	White	Indian
**n**	2052	668	397	440	2996	997	492	552	3498	465	156	40
**Age – mean years (95% CI)**	28.5 (27.7–29.3)	29.6 (28.1–31.2)	32.6 (30.8–34.5)**	32.3 (30.6–34.0)**	27.8 (27.2–28.3)	28.9 (28.0–29.8)	30.4 (29.0–31.8)**	29.2 (27.9–30.5)	28.2 (27.7–28.7)	30.6 (29.2–32.0)**	31.8 (29.3–34.2)**	31.9 (29.6–34.3)**
**Ever had sex - % (95% CI)**	79.4 (76.3–82.5)	83.1 (76.4–88.2)	79.8 (76.5–82.7)	80.0 (74.0–84.9)	80.2 (77.7–82.4)	77.6 (73.1–81.6)	79.4 (73.3–84.3)	66.0 (54.5–75.9)**	87.2 (85.7–88.6)	84.7 (78.2–89.6)	78.6 (66.4–87.2)	91.5 (83.0–95.9)
**Age of debut - mean years (95% CI)**	18.1 (17.9–18.4)	18.5 (18.0–19.1)	19.1 (18.3–19.8)*	19.9 (19.0–20.7)***	18.0 (17.7–18.2)	18.1 (17.8–18.5)	19.4 (18.9–20.0)***	19.8 (19.0–20.5)***	17.1 (16.9–17.3)	17.8 (17.1–18.4)*	18.3 (17.6–19.0)***	17.7 (16.8–18.7)
**% with more than one sex partners in last 12 months (95% CI)** a	20.0 (15.4–25.5)	15.6 (8.2–27.7)	3.0 (1.3–6.9)	4.9 (2.3–10.1)	13.7 (11.7–16.0)	8.8 (6.7–11.3)**	2.4 (1.4–4.2)***	2.5 (1.3–4.7)***	24.2 (21.7–26.8)	11.2 (7.1–17.1)**	4.1 (1.3–12.0)**	5.9 (1.0–27.1)
**Mean/median no. sex partners in last 12 months (95% CI)** a	1.38/1 (1.23–1.52)	1.24/1 (1.11–1.37)	1.05/1 (1.01–1.09)***	1.07/1 (1.02–1.11)***	1.42/1 (1.32–1.52)	1.34/1 (1.21–1.46)	1.13/1 (1.00–1.26)***	1.14/1 (0.96–1.32)**	1.4/1 (1.3–1.4)	1.2/1 (1.1–1.3)***	1.2/1 (0.9–1.3)**	1.3/1 (0.8–1.7)
**Age gap -% (95% CI)** a **^,^** b	0.6 (0.3–1.5)	9.0 (5.2–15.2)***	3.2 (1.0–10.2)	0.6 (0.1–2.3)	0.3 (0.1–0.6)	1.3 (0.7–2.4)***	1.5 (0.4–5.2)	1.1 (0.3–3.5)	1.0 (0.7–1.5)	3.0 (1.5–5.8)**	1.4 (0.5–3.9)	2.9 (0.4–19.6)
**Concurrency - % (95% CI)** a	8.6 (6.6–11.3)	6.0 (2.0–16.4)	0.5 (0.1–1.6)***	1.7 (0.7–4.7)***	10.8 (9.1–12.7)	5.5 (3.7–8.1)**	0.9 (0.3–2.3)***	1.7 (0.8–3.7)***	14.6 (12.8–16.6)	5.3 (2.9–9.4)***	2.3 (0.6–8.8)***	3.6 (0.5–23.2)
**Condom use - % (95% CI)** a	65.8 (60.1–71.2)	53.4 (42.8–63.6)*	44.2 (30.3–59.1)**	37.6 (27.2–49.3)***	38.3 (35.1–41.5)	21.7 (18.0–26.0)***	19.6 (15.3–24.6)***	29.1 (22.4–36.9)	58.1 (54.7–61.5)	35.8 (26.9–45.9)***	15.7 (9.5–24.9)***	30.8 (12.5–58.0)
**Circumcision - % (95% CI)^e^**	39.5 (35.3–43.8)	20.5 (15.0–27.3)***	33.1 (22.4–45.9)	22.6 (16.8–29.8)***					48.4 (45.1–51.7)	23.1 (16.5–31.5)***	16.3 (9.4–26.5)***	29.6 (18.1–44.5)
**Women**												
**n**	2503	800	438	454	4727	1398	683	730	3934	635	217	81
**Age – mean years (95% CI)**	29.1 (28.5–29.6)	31.5 (30.4–32.7)***	32.7 (31.3–34.3)***	31.1 (29.8–32.3)**	29.5 (29.2–29.8)	30.6 (29.8–31.4)**	33.6 (32.7–34.4)***	31.6 (30.1–33.1)**	29.5 (29.1–29.8)	32.4 (31.4–33.4)***	34.4 (32.8–35.9)***	33.2 (31.0–35.4)**
**Ever had sex - % (95% CI)**	83.2 (80.7–85.5)	89.5 (86.2–92.0)**	82.4 (73.8–88.6)	70.9 (63.9–77.0)***	86.8 (85.3–88.1)	83.6 (80.6–86.1)	85.5 (81.3–88.8)	75.2 (67.6–81.5)***	91.0 (89.9–92.0)	90.8 (86.8–93.7)	92.7 (87.5–95.9)	83.4 (68.9–91.9)
**Age of debut - mean years (95% CI)**	18.0 (17.9–18.2)	19.0 (18.5–19.6)**	20.1 (19.5–20.7)***	20.2 (19.8–20.7)***	18.1 (17.9–18.3)	18.8 (18.5–19.2)***	20.3 (19.7–20.8)***	19.5 (18.4–20.7)*	17.9 (17.8–18.1)	18.5 (18.0–19.0)*	18.7 (17.9–19.5)	20.3 (19.5–21.5)***
**% with more than one sex partners in last 12 months (95% CI)** a	4.3 (3.0–6.1)	3.0 (1.4–6.1)	1.8 (0.7–4.7)	0.6 (0.2–2.0)***	1.8 (1.5–2.4)	2.5 (1.5–4.0)	2.5 (0.9–6.8)	0.08 (0.00–0.5)***	3.6 (2.8–4.7)	4.8 (2.6–8.8)	0.02 (0.00–0.1)***	0 (0.0–0.0)
**Mean/median no. sex partners in last 12 months (95% CI)** a	1.08/1 (1.03–1.14)	1.08 (0.98–1.19)	1.05/1 (0.99–1.10)	1.01/1 (1.00–1.02)**	1.11/1 (1.03–1.20)	1.21/1 (0.99–1.43)	1.56/1 (0.52–2.59)	1.00/1 (0.99–1.00)*	1.04/1(1.03–1.05)	1.07/1 (1.01–1.13)	1.00/1 (1.00–1.00)***	1.00/1 (1.00–1.00)***
**Age gap -% (95% CI)** a **^,^** b	47.3 (43.0–51.5)	33.4 (25.9–41.8)**	19.1 (11.2–30.6)***	25.1 (15.3–38.5)**	13.8 (12.3–15.5)	17.1 (12.9–22.2)	13.1 (8.9–18.8)	25.2 (15.0–39.1)	29.8 (28.1–31.7)	20.6 (16.8–25.0)***	27.0 (19.6–36.0)	28.5 (21.2–37.2)
**Concurrency -% (95% CI)** a	1.0 (0.5–2.1)	0.7 (0.2–2.3)	0.8 (0.2–2.3)	0.0 (0.0–0.0)	1.2 (0.9–1.7)	0.9 (0.4–2.3)	0.7 (0.2–2.2)	0.5 (0.2–1.9)	1.2 (0.7–1.7)	0.7 (0.1–4.0)	0	0
**Condom use -% (95% CI)** a	65.7 (59.7–71.2)	33.3 (23.4–44.9)***	24.5 (14.9–37.6)***	31.8 (20.1–46.3)***	32.2 (30.0–34.5)	12.4 (9.0–16.6)***	16.5 (12.0–22.3)***	8.1 (6.1–10.7)***	41.2 (38.5–43.9)	24.8 (18.8–32.0)***	19.1 (12.3–28.4)***	20.6 (10.6–36.4)**

* *P*<0.01, **P<0.001, ***P<0.0001 (The *P*-values refer to comparisons between the each racial group and the black group within that same survey.

a These analyses are limited to the individuals who are sexually experienced.

b Age-gap refers to the respondents most recent sexual partner being 5 or more years older than the respondent.

The only exception was the DHS 2003 survey where this gap was measured as 10 years.

### Sexual debut

Among men, the mean age of sexual debut was significantly lower in the black group than in the white and Indian groups in all the surveys. There was no significant difference between the coloured and black groups in the 2003 DHS, 2002 and 2005 SABSSM. In the case of the CAPS and the NCS, the age of debut for the coloureds was significantly later than that for the blacks but earlier than that for the whites. For women, the age of sexual debut was significantly lower in the black group than the other groups in all the surveys with two exceptions. For the coloureds in the 2003 DHS and the whites in the NCS, sexual debut occurred at a non-significantly later age than the black women (*P* – 0.07 and 0.05 respectively).

### Sexual experience

In both sexes, the proportion of individuals who had experienced sexual intercourse did not differ significantly between the races, with three exceptions. Firstly in the CAPS, which was the only survey limited to young persons, the proportion who had had sex was significantly higher for the blacks than the other groups. For women, 89.7% (95% Confidence Interval (CI), 86.7–92.0) of blacks versus 59.9% (95% CI, 55.7–64.0) of coloureds and 57.4% (95% CI, 49.1–65.3) of whites had had sex. For men these percentages were 86.2% (95% CI, 83.5–88.4) for blacks, 66.8% (95% CI, 62.5–70.9) for coloureds and 49.5% (95% CI, 39.8–59.1) for whites. The second exception was the significantly lower proportion of Indian women reporting sexual experience. This was the case for the 2003 DHS, the 2002 and 2005 SABSSMs and there was a trend in this direction in the NCS (*P*-0.08). In only one of the four surveys where this was assessed, did a significantly lower proportion of Indian men report having had sex. This was in the 2005 SABSSM. Thirdly, in the 1998 DHS, a significantly higher percentage of the black women (88.5%, 95% CI, 87.5–89.3) reported having had sex than the coloured, white and Indian women (81.2%, 95% CI, 78.5–83.7; 80.6%, 95% CI, 76.4–84.3 and 75.2%, 95% CI, 71.1–78.9 respectively).

### Partner age gap

For men, the coloured group had the highest prevalence of partners five or more years older than the respondent. Although absolute numbers were not high, in all three of the 15–49 year old surveys with available data the coloured men had a significantly higher prevalence of older partners than the black men. In the CAPS there was no association found. Among women, the blacks had the highest prevalence of older partners, excluding the 2003 DHS, where the whites had a non-significantly higher prevalence. Prevalence in the black women was significantly higher than the whites in the CAPS, the coloureds in the NCS and 2005 SABSSM and all three other racial groups in the 2002 SABSSM.

### Number of sex partners

For the men in all five surveys, the black group had the highest proportion of individuals who had had more than one sexual partner in the past 12 months. This proportion was significantly higher than all other racial groups in all the surveys. The only exceptions were in the case of the coloureds in the 2002 SABSSM and the Indians in the NCS where the trends were in the same direction but not statistically significant. In the five surveys, the proportion of men with more than one sex partner varied from three- to six-fold higher in the blacks than the other racial groups. The black men also had a higher mean number of sexual partners in the past 12 months than the other groups. The mean for the black men was significantly elevated versus the whites in all the surveys, significantly elevated versus the Indians in the 2002 and 2005 SABSSM and non-significantly in the NCS and 2003 DHS. The mean for the coloured men was significantly lower than that of the black men in the NCS and non-significantly lower in the other surveys. In all the surveys the mean for the coloured men was intermediate between that of the black and white men.

In the case of the women, there was little evidence of a covariance between racial HIV prevalence and number of sexual partners. The groups with the highest proportion of individuals with more than one partner in the past year were the whites in the CAPS, the coloureds in the NCS, both these groups in the 2005 SABSSM and the blacks in the 2002 SABSSM and the 1998 and 2003 DHSs. In all five surveys with data, the Indian women had a significantly lower proportion with more than one partner than the black women.

The CAPS was the only survey with data on the lifetime number of sexual partners. There was no evidence of a variation between race and this variable in this survey. In blacks, coloureds and whites, the mean number of lifetime partners in this survey of young persons was 2.9 (95% CI, 2.7–3.1), 2.7 (95% CI, 2.3–3.1) and 2.6 (95% CI, 1.5–3.6) in men and 2.3 (95% CI, 2.2–2.4), 1.5 (95% CI, 2.2–2.4) and 2.4 (95% CI, 2.2–2.4) in women, respectively.

### Concurrency

In the case of the men, the black group had higher self-reported concurrency prevalences than the other groups in all five surveys. The concurrency prevalence in the black group varied from 7 to 16 times higher than that of the whites in the different surveys. In all the surveys the coloured men had an intermediate prevalence of concurrency (between that of the whites and blacks). In the case of the women, self-reported concurrency prevalences were highest in the black group in all five surveys with available data, but this relationship was only statistically significant in the CAPS. In the CAPS, the cumulative concurrency prevalence in the black women (15.9%, 95% CI, 13.4–18.7) was considerably higher than that for the coloureds (1.7%, 95% CI, 0.9–2.8%) and whites (2.7%, 95% CI, 1.0–7.2). In the 1998 DHS, concurrency in those who were married was higher in the blacks (7.5%, 95% CI, 6.4–8.6) than the whites (4.1%, 95% CI, 2.5–6.4) and Indians (0.5%, 95% CI, 0.1–1.9) but not the coloureds (6.9%, 95% CI, 5.2–9.1).

### Condom usage

Condom use at last sex was most prevalent amongst the black group for both men and women. The only exceptions to this were the women in CAPS and the men in the 2003 DHS where the white groups had non-significantly higher condom usage rates than the black groups.

### Circumcision

The prevalence of self-reported circumcision was highest amongst the black group in all three surveys that collected data on this. Typically prevalences were more than twice as high amongst the blacks than the other racial groups.

## Discussion

Over 30 years into the HIV epidemic there is still little consensus as to what drives the generalized HIV epidemics in sub-Saharan Africa [9], [10]. The large differences in HIV prevalence between the various races in South Africa offer a useful standpoint from which to investigate putative risk factors. South Africa has conducted three nationally representative HIV serosurveys that include 15–49 years olds. In 2004, the HIV prevalence in 15–49 year olds was 19.9% (95% CI, 18.1–21.4) in blacks, 3.2% (95% CI, 2.1–4.3) in coloureds, 0.9% (95% CI, 0.08–1.7) in Indians and 0.5% (95% CI, 0.1–0.9) in whites. HIV prevalences by race vary to a similar degree in the other two surveys conducted in 2001 [7] and 2007 [11] as well as in a nationally representative sample of 15–24 year olds [12], a national survey of tertiary students [13], a survey of company employees [14] and the country's annual antenatal surveys [15]. Controlling for various socioeconomic variables makes little or no difference to the differences in HIV prevalence by race [12], [15], [16]. An example is provided by a multivariate analysis of the 2004 HIV survey. When education and socioeconomic status are controlled for, being black remains the strongest factor associated with testing HIV positive – the odds ratios varying from 7.9 (95% CI, 4.3–14.5) to 8.7 (95% CI, 5.1–14.8) in the men and women only models respectively [17].

There has only been one published study that has attempted to systematically explore the risk factors which co-vary with HIV prevalence by race in South Africa [2]. This study found that the individual-level risk factors such as number of sex partners in the last 12 months, condom usage and circumcision prevalence did not co-vary with HIV prevalence by race. The prevalences of partner and respondent concurrency, both network-level properties, were however found to differ considerably between the different racial groups and to do so in a way which mirrored the differences in HIV prevalence. This study was limited to 14–22 year olds in the city of Cape Town. The current study extends this analysis to include five nationally representative samples of 15–49 year olds.

Its findings concur to some extent with the Cape Town study. The Indian and white groups are both numerically small and have similarly low HIV prevalences. If we consider them together as the low HIV prevalence groups, then the risk factors which co-vary with HIV prevalence by race in the six surveys are age of sexual debut (in five out of five surveys for men and three out of six surveys for women), age gap (zero surveys in men and three in women), mean number of sex partners in the previous year (five surveys in men and one in women) and respondent concurrency (five surveys in men and one in women). Condom usage and circumcision were both more prevalent in the high HIV prevalence groups. There was little evidence of difference in the prevalence of those who had had sex. The survey which demonstrated the largest difference in this variable was the CAPS. This was likely due to the fact that it was the only survey which was limited to younger persons. In four of the five surveys where people up to the age of 49 were included, there were no differences in sexual experience.

Which of the co-varying risk factors could be responsible for the large differential HIV spread by race? Age of sexual debut, by itself, is an unlikely candidate. This is for a number of reasons, including the fact that the age of sexual debut in the highest HIV prevalence groups is higher than that in the very low prevalence countries of the USA [18] and Western Europe [19].

A number of publications have argued that age-mixing plays a significant role in HIV spread in sub-Saharan Africa [20], [21], [22]. Age-mixing, whilst of likely importance, cannot without an interconnected sexual network result in a generalized HIV epidemic. This is evident if we consider a hypothetical population where there is an age gap of 10 years in all couples but the couples practice exclusive lifetime monogamy. Purely sexually transmitted infections cannot spread in this population despite extreme age-mixing since STI spread depends on an interconnected sexual network [23]. Factors such as age-mixing are, however, likely to influence transmission across an interconnected network, particularly to new cohorts of younger persons [22], [24]. The fact that, in three out of five surveys of women, the prevalence of age-discordant coupling co-varied with HIV prevalence may be indicative of age-mixing having an influence on HIV prevalence.

This analysis finds evidence of a covariance between concurrency and HIV prevalence. Higher prevalences of sexual partner concurrency have been shown to lead to exponential increases in the degree of network connectivity and thereby the potential for HIV transmission [25]. Although certain studies have not found an association between HIV and concurrency [26], [27], a number of good modeling-based and empirical studies have shown that concurrency prevalence covaries closely with HIV prevalence inter- and intra-nationally [2], [23], [28], [29] and that it is a key driver of incident HIV at a partnership level in sub-Saharan Africa [30]. In particular declines in concurrency have been shown to be important in the rapid decline of HIV incidence in Zimbabwe, Uganda and elsewhere [31], [32]. Amongst the women, the prevalence of concurrency was only higher in the blacks in one of five surveys – the CAPS survey. Finding lower prevalence of concurrency in women compared to men is a common result of surveys in Southern Africa and further afield [2], [23], [28], [33], [34], [35], [36]. This may reflect a combination of a lower prevalence of concurrency [34] and a differential male-female courtesy bias induced by the fact that concurrent partnering is considerably more stigmatized for women than men in many communities [37]. The importance of a courtesy bias in this regard is suggested by studies in Southern Africa that found that changes in the ways that surveys are conducted and the ways questions are asked, can lead to a significant increase in the measured prevalence of concurrency in women [33], [38]. Even in the likely scenario that women are less likely to have concurrent partners than men, concurrency can still lead to extensive HIV spread in women. This is for two main reasons. Firstly, at an individual level, concurrency acts to increase the risk of HIV to the partners of the individual engaging in concurrency rather than to the individual him or herself [23], [24], [28]. Secondly, and most importantly, concurrency's major impact on HIV transmission operates by connecting together a large proportion of the population into a transmission pathway for HIV [28], [36]. This is a network level effect and thus would be experienced by all members of the connected-network (both men and women).

The total number of sexual partners, though important, is less likely to be a crucial factor for a number of reasons. Firstly, the lifetime number of partners is no higher in sub-Saharan African countries with generalized HIV epidemics than the USA [18] and other low HIV prevalence countries [19], [23]. Secondly, the available evidence suggests that much of the higher number of sexual partners in the past year amongst the black group represents long-term concurrent partnering [2], [23]. This is supported by the fact that this analysis could find no evidence of a difference in the total life-time number of partners between the races.

Other comparative studies of sexual behavior in sub-Saharan Africa have reached different conclusions. In a comparative study of sexual behavior of 18–24 year olds in the USA and South Africa, Pettifor et al, found that three out of four risk factors assessed (age of sex-debut, lifetime number of sex partners and lack of condom usage) were more prevalent in the USA (HIV prevalence <1%) than South Africa (HIV prevalence 10.2%) [18]. Only age-mixing was more prevalent among South African women. Pettifor et al, reached the conclusion that “unique biological forces” must be important factors in explaining the more extensive spread of HIV in South Africa. Of note, this study did not assess differences in concurrency prevalence [39].

## Limitations

There are a number of limitations that apply to this study. The surveys used were based on interviews about sensitive topics which were generally conducted in the respondents' residences, often with other individuals within listening distance. In addition some of the surveys had low response rates. The data is thus susceptible to a large number of biases such as courtesy, recall and non-response biases. There is however little evidence that we are aware of that there is a difference in sexual behaviour between those who do and do not answer sexual behavior questionnaires [40]. This is important as there was evidence of a differential response rate by race in some of the surveys. This was most marked in the CAPS where the Wave 3 response rates varied from 71% in the blacks to 57% in the whites. If responders varied from non-responders by sexual behavior then this could confound our results. It is likely that sensitive information such as the extent and type of multiple-partnering is underreported, particularly among women [34]. There was however no evidence we could find of a differential bias by racial, ethnic or national group in this or any other sexual behavior topic. Since the comparisons were between racial groups within each survey, this type of underreporting should not invalidate our comparisons.

“It must be borne in mind that HIV prevalence at a point in time represents the cumulative effect of behaviours over the preceding decade and longer [41]. Differences in current HIV prevalence therefore reflect the cumulative effect of behaviours over the preceding years. Behaviors may have changed over this time and they may have changed in response to the HIV epidemic. The surveys reviewed here, do however, span a period from 1998–2009 and there is little evidence of a change in the variance of the risk factors by race over this period. As an example the only longitudinal study that we are aware of that has reported changes in the prevalence of concurrency in South Africa has found that the difference in concurrency prevalence between black and coloureds has not changed between the times it was measured (2005–2009) [42]. The clearest example of a change is the increase in condom usage, which is likely a response to the HIV epidemic [11]. If we compare condom use at last sex between the earliest survey (DHS 1998) and the last survey (NCS 2009), condom usage has increased in all groups. The initial prevalence of condom usage in the black women (no men were surveyed in 1998) was however higher (12.6%) than the coloureds, whites and Indians (7.9, 9.6 and 6.5% respectively). The black women were also the group with the greatest absolute increase in condom usage – by 2009 the prevalence of condom use at last sex was 41.2, 24.8, 19.1 and 20.6% for the black, coloured, white and Indian women respectively. Neither the 1998 nor the 2009 results are therefore compatible with the thesis that differences in condom use are a key reason for differences in racial HIV prevalence.”

The analysis is explicitly ecological in nature thus making any inferences to the individual level inappropriate. The relationship between race, concurrency and HIV prevalence may be confounded by other unmeasured variables. Biological differences or differences in the prevalence in other risk factors for HIV susceptibility and transmission such as bacterial vaginosis have been put forward as explanations of racial differences in HIV prevalence [43]. This study is unable to directly assess these hypotheses. However, both in South Africa and the USA, the racial groups with highest HIV prevalences also have higher prevalences of the other major STIs [6], [28], [44]. It is more likely that the raised STI prevalences in particular groups are due to the behavioral risks (such as concurrency) which co-vary with the rates of all the major STIs than that each STI has a biological vulnerability associated with the same racial groups [28], [44]. The evidence presented here is that the differences in sexual behavior in general, and the prevalence of multiple partnering in particular, could explain, in a fairly parsimonious fashion, at least a part of the large differences in HIV prevalence between South Africa's racial groups.
